# A Network Visualization Query System for Multidrug Compatibility Based on a WeChat Mini Program: Preliminary Usability and Efficiency Evaluation

**DOI:** 10.2196/86583

**Published:** 2026-07-21

**Authors:** Jianhui Yang, Xiaoqing Huang, Shurong Wu, Yuanyuan Zhang, Xiaofang Zhuang, Yilun Dong, Biru Li, Zhida Hong

**Affiliations:** 1 Department of Pharmacy, Women and Children’s Hospital, School of Medicine, Xiamen University, No.10, Zhenhai Road, Siming District, Xiamen, Xiamen, China, 86 13225928281

**Keywords:** drug compatibility, network visualization, user experience, weChat, drug interactions, drug safety, medication safety

## Abstract

**Background:**

Intravenous drug incompatibility is a significant medication safety hazard, particularly in complex multidrug regimens. Traditional text-based, pairwise query methods are inefficient and impose a substantial cognitive load on clinicians. While network visualization has the potential to address these challenges, its application in drug compatibility queries remains underexplored.

**Objective:**

This study aimed to design, develop, and evaluate a novel drug compatibility query system based on a WeChat Mini Program. The system integrates diverse data sources and uses network visualization to present complex compatibility relationships. We sought to empirically assess its impact on efficiency and user experience.

**Methods:**

A preliminary crossover usability and efficiency evaluation was conducted. Phase 1 involved the construction of a drug compatibility knowledge base from authoritative handbooks and drug labels, and the development of the query system. Phase 2 comprised a system evaluation with 36 pharmacists, 6 physicians, and 5 nurses. The evaluation included a scenario-based task completion time analysis comparing the system (Mode A) with traditional print-based resources (Mode B), a quality assessment using the Mobile Application Rating Scale (MARS), and structured posttask user feedback to gather insights on user experience.

**Results:**

The query system demonstrated a substantial reduction in task completion time, with median time savings of 2.85 (IQR 1.98‐4.15) minutes, 5.45 (IQR 3.95‐7.20) minutes, and 31.2 (IQR 27.5‐35.1) minutes, respectively, in 3 scenarios with different complexity. The system received a high mean overall quality score of 3.88 (SD 0.35) on the MARS. The functionality dimension scored the highest (mean 4.21, SD 0.51), while engagement scored the lowest (mean 3.21, SD 0.62). Posttask structured feedback revealed five major areas of user feedback: (1) baseline experience and first impressions, (2) user experience with network visualization, (3) perceived efficiency and cognitive load, (4) trust and information quality, and (5) future application considerations. Users praised the system’s efficiency and intuitive design but expressed a strong need for transparent data sources and management advice to build trust.

**Conclusions:**

A query system based on network visualization demonstrates potential to support the efficiency of multidrug compatibility queries within this preliminary evaluation. It may mitigate cognitive load and offer an at-a-glance understanding of complex drug relationships. However, formal clinical accuracy validation remains a mandatory precondition before bedside clinical deployment is considered.

## Introduction

Medication safety is a critical issue in global health care. Intravenous (IV) administration, as one of the most common routes of drug delivery, raises significant safety concerns that directly impact patient survival. IV therapy is associated with numerous medical risks, among which drug incompatibility remains a substantial safety hazard [1,2].

Drug incompatibility is defined as a phenomenon wherein 2 or more drugs, when mixed in vitro, undergo physical or chemical reactions that alter their properties. According to the reaction mechanisms, drug incompatibility can be classified into two groups: physical incompatibility and chemical incompatibility [3]. Drug incompatibilities can lead to the occlusion of IV lines and, more seriously, can cause life-threatening complications such as pulmonary embolism and organ damage due to the formation of microparticulates [4,5]. Moreover, chemical degradation directly results in therapeutic failure and the generation of potentially toxic new substances [6].

Many studies have confirmed that drug incompatibilities are frequently encountered in clinical practice, particularly in intensive care units (ICUs), where medication regimens are complex and vascular access is limited. Up to 7.2% of all theoretical drug pairings in an adult ICU were revealed to be incompatible [7], and nearly 85% of pediatric patients were exposed to at least one potential incompatibility event during their hospitalization [8]. This poses considerable decisional pressure and safety risks for health care professionals working in high-intensity environments [9].

A variety of reference tools have been developed to address these risks. Trissel’s Handbook on Injectable Drugs is widely recognized as the “gold standard” in drug compatibility, while online databases such as Micromedex and Lexicomp offer timely updates and convenient searchability. However, their fundamental design, reliant on text-based, pairwise queries, creates a significant bottleneck in complex clinical scenarios. This method forces clinicians to manually check each drug pair one by one, a process that is not only inefficient but also imposes a substantial extraneous cognitive load, especially when assessing multidrug regimens for patients with critical illness [10]. Another challenge is a significant lack of compatibility data. One study revealed that physical compatibility data for drug combinations commonly used in ICUs were missing for as high as 46% of combinations, while chemical compatibility data were absent for 93% [11]. Additionally, inconsistent conclusions on drug compatibility exist across different resources [12,13], forcing clinicians to cross-reference multiple sources. The English-only format of these tools also limits their use in resource-constrained primary health care settings in non-English-speaking countries [14].

A paradigm shift from linear text searches to holistic data representation is required to address this core challenge of cognitive overload. Studies on electronic health record (EHR) visualizations provide compelling evidence that well-designed visual interfaces can significantly reduce cognitive load and enhance decision-making efficiency [15-17]. By translating complex, multirelational data into intuitive graphical representations, visualizations support safer and faster clinical judgments. Network visualization, in particular, is theoretically well-suited to the multidrug compatibility problem, as it can simultaneously display all relationships within a drug regimen, intuitively highlight incompatibilities, and clearly demarcate areas of missing data.

While some health care institutions have developed localized charts or tables to enhance clinical applicability [18,19], a more dynamic and comprehensive solution is needed to fundamentally address the challenges of cognitive load and data complexity. Accordingly, the objective of this study was to design, develop, and conduct a preliminary assessment of a novel drug compatibility query system. This system, based on the WeChat Mini Program, integrates diverse data sources and uses network visualization to intuitively present complex compatibility relationships. The study further aimed to empirically assess the system’s impact on efficiency and user experience through a preliminary crossover evaluation and posttask structured feedback, in order to validate its potential for reducing cognitive load and improving medication safety in clinical practice.

## Methods

### Overview

This study followed a usability and efficiency evaluation accompanied by posttask structured feedback and was divided into 2 phases from March 2023 to September 2025. The study was assessed against the CONSORT (Consolidated Standards of Reporting Trials) extension guidelines. Phase 1 involved the construction of a drug compatibility knowledge base and the development of a network visualization system for multidrug compatibility queries based on WeChat Mini Program. In phase 2, the efficiency and quality of the newly developed query system were assessed.

### Phase 1 System Development (From March 2023 to April 2025)

#### Knowledge Base Construction

To build a comprehensive drug compatibility knowledge base, this study integrated data from drug labels currently used with 2 authoritative handbooks. While drug labels are legally mandated, they often lack complete compatibility information due to the inability of premarketing trials to assess all drug compatibilities and the significant delays in updating labels with postmarket findings. Therefore, the *432 Injectable Drugs Compatibility Handbook* [20] and *ASHP’s Handbook on Injectable Drugs* [21] (which was the most recent and widely adopted version in our hospital during the initial knowledge base construction) were used as supplements. The *432 Injectable Drugs Compatibility Handbook* (Chinese Handbook) is a common reference for drug compatibility in China and is recommended by *Expert Guidance for the Safe Administration of Intravenous Drugs* issued by the Guangdong Pharmaceutical Association [22]. *ASHP’s Handbook on Injectable Drugs* (ASHP’s Handbook) is recognized as the global gold standard for compatibility information [12,19] and is widely used as a benchmark for clinical practice and research due to its evidence-based content [12,23,24].

### Data Extraction and Standardization

Data were extracted using a standardized form capturing key fields, including drug combination details (name, manufacturer, and concentration), solvent, compatibility result, experimental conditions (eg, temperature, contact duration, and light exposure), container type, and data source. To accommodate variability among the sources, not all fields were mandatory for each entry. To ensure the accuracy and reliability of the knowledge base, data extraction was performed independently by 2 clinical pharmacists (JHY and XQH) for the entire dataset. Any discrepancies were resolved through a third senior clinical pharmacist (ZDH).

We implemented a protocol to ensure data consistency. Drug names were standardized using the international nonproprietary name (INN). All drugs were classified by the World Health Organization (WHO) Anatomical Therapeutic Chemical (ATC) System. All terms from drug labels implying incompatibility (eg, “do not,” “prohibited,” “not recommended”) were uniformly classified as “Incompatible.”

Particularly, if a drug pair had conflicting compatibility results across different references, the system strictly defaulted to a red edge to maximize safety alerts.

### System Design and Development

The drug compatibility query system was developed as a WeChat Mini Program using WeChat Developer Tools (Stable 1.06.2209190). The user interface was built using WeiXin Markup Language (WXML) and WeiXin Style Sheets (WXSS), which are adaptations of HTML and CSS. The core application logic was programmed in JavaScript. A Cloud database, a JSON-based document database similar to MongoDB, was used to store the structured compatibility data. The ECharts.js library was used to construct a network graph for visualizing multidrug compatibility relationships.

### Phase 2 System Evaluation (From May 2025 to September 2025)

#### Participants

To ensure diverse perspectives, licensed pharmacists actively working in PIVAS, ICUs, or inpatient wards across several medical institutions in Xiamen, China, were identified from departmental rosters and approached individually through professional networks and direct invitations. Physicians and nurses working in the same clinical environments (eg, ICUs and pediatric wards) were also purposively approached and invited directly through professional networks. Purposive sampling criteria for all participants required at least one year of clinical work experience and familiarity with smartphone use.

### Evaluation Procedures

The evaluation consisted of 3 parts: a scenario-based task completion test, a standardized quality rating via the Mobile App Rating Scale (MARS), and a semistructured interview.

#### Scenario-Based Task Completion Time Analysis

All pharmacists participated in the task completion time analysis. We designed 3 task scenarios that pharmacists commonly received consultation from diverse clinical departments. These scenarios ranged from a simple pairwise compatibility query to a complex multidrug regimen optimization and were designed to ensure a comprehensive evaluation of the system’s utility in real-world clinical practice. To enhance their validity, these scenarios were reviewed and confirmed for appropriate complexity by two senior clinical pharmacists not involved in the study.

Scenario 1: a patient in the department of Breast and Plastic Surgery had a central venous catheter (CVC) placed. In order to prevent catheter-related bloodstream infections (CRBSIs), the nurse was planning to use a vancomycin-heparin lock solution. Is this combination rational?

Scenario 2: the patient was a fluid-restricted premature neonate with limited venous access. The patient’s sole access was a multilumen peripherally inserted central catheter (PICC). Ongoing infusions included parenteral nutrition (PN) via a dedicated lumen, a continuous co-infusion of dopamine (vasoactive agent) and midazolam (sedative), and an intermittent infusion of vancomycin which had just been completed. The primary clinical challenge was the safe administration of a newly prescribed intermittent infusion of ampicillin, and how to integrate the new antibiotic without compromising existing therapies.

Scenario 3: a critically ill child in the PICU has a single, triple-lumen CVC but requires the simultaneous infusion of six drugs: midazolam, fentanyl, vancomycin, dopamine, norepinephrine, and furosemide. The nurse must decide which drug infusion lines can be safely connected via Y-sites into the available lumens. Please provide a rational infusion regimen.

The task execution involved 2 query modes. In mode A, participants used our newly developed query system, while in mode B, they used three print-based data sources: drug labels, the Chinese handbook, and ASHP’s handbook. These 3 paper-based references represent the most authoritative and readily accessible sources of compatibility information currently available in daily clinical practice. Prior to the development of our system, pharmacists relied heavily on manually cross-referencing these specific paper-based volumes as their primary reference standards when resolving complex bedside compatibility queries. Although established international electronic databases (eg, Micromedex or Lexicomp) exist, they are not widely used in local routine practice due to high institutional subscription costs. Therefore, these selected paper-based resources represent the most realistic and authoritative standard of care available in this setting.

Participants were randomly assigned to complete the tasks first (via coin toss) in one mode and then in the other. Task completion time was recorded, and a 30-minute break was scheduled between the 2 modes.

#### MARS Completion

After performing queries with the query system, all of the pharmacists participated in the MARS rating to assess the system’s quality. All pharmacists received MARS training by watching videos [25] prior to the assessment. The MARS is a widely recognized, reliable, and innovative multidimensional assessment tool developed by Stoyanov and his team [26], specifically designed to systematically evaluate the quality of mobile health (mHealth) apps such as pharmacy apps [27-29]. There are 23 assessment items in MARS, which are categorized into four objective quality dimensions (engagement, functionality, aesthetics, and information quality), and one subjective quality dimension. Each item uses a 5-point Likert scale (from 1=Inadequate to 5=Excellent), with a “Not Applicable” (N/A) option provided to enhance flexibility.

#### Qualitative Feedback With Semistructured Interviews

Following the quantitative evaluation, semistructured interviews (approximately 5‐10 min) were conducted face-to-face with the 32 health care professionals (22 pharmacists, 5 physicians, and 5 nurses). The interview guide (Multimedia Appendix 1) mainly explored the system’s strengths, weaknesses, user experience, and suggestions for improvement. Interviews were audio-recorded, transcribed, and anonymized for analysis.

### Statistical Analysis

Continuous variables were reported as mean (SD) for normally distributed data and as median with IQR for non-normally distributed data. Differences in task completion time between the 2 query modes (mode A and B) were analyzed using Wilcoxon signed-rank tests across the 3 scenarios. To account for multiple comparisons across the 3 scenarios, a Bonferroni correction was applied, setting the significance threshold at α=.0167 (0.05/3). To assess potential carryover or learning effects inherent in the crossover design, a Wilcoxon rank-sum test (Mann-Whitney *U* test) was conducted to compare Mode A completion times between the two sequence groups (Sequence 1: Mode A followed by Mode B; Sequence 2: Mode B followed by Mode A) across all scenarios, with a *P* value greater than .05 indicating no significant order effect. The internal consistency of the MARS subscales was assessed using Cronbach α coefficients, interpreted as poor (<0.50), acceptable (0.50‐0.75), good (0.75‐0.90), or excellent (>0.90). Furthermore, Spearman rank correlation analysis was performed to evaluate the relationships between user demographics (age, education level, and work experience), objective efficiency gains (time saved), and subjective usability ratings (MARS scores). All statistical analyses were conducted using R software (version 4.3.1; R Foundation for Statistical Computing).

Posttask structured feedback was transcribed and descriptively analyzed. Two researchers, XQH (a female junior pharmacist) and BRL (a male senior pharmacist), independently categorized the feedback into user-reported strengths, weaknesses, and suggestions using NVivo (Release 15.0.0; Lumivero). A preexisting collegial relationship existed between some pharmacist participants and the interviewers. We addressed this potential bias through proactive reflexivity, specifically by maintaining reflexive journals to document preconceptions and conducting peer debriefing with an independent researcher to verify the findings. The resulting codes were then collaboratively grouped into key descriptive categories. Any discrepancies were resolved through discussion with a third researcher with no preexisting collegial relationships with the participants to reach consensus. Furthermore, member checking was performed with a subset of participants to ensure that the interpreted description accurately reflected their original perspectives. Key descriptive categories were illustrated with direct, anonymized quotes.

### Ethical Considerations

Our study protocol was conducted in accordance with the principles of the Declaration of Helsinki and approved by the Research Ethics Committee of the Women and Children’s Hospital, School of Medicine, Xiamen University (KY-2025‐105 K01). All participants were told the study content and could withdraw at any time, and individual data were deidentified. Informed consent was obtained from all participants before data collection. The study was conducted in 2 stages: Phase 1 (March 2023-April 2025) was dedicated to database and software development, which did not involve human participants and thus did not require ethics approval; Phase 2 (commencing May 2025) involved human participants for system evaluation, for which ethics approval was obtained prior to any data collection.

## Results

### Knowledge Base Content

A list of 212 injectable drugs in use at our hospital was constructed, representing 204 distinct active ingredients. Anti-infectives for systemic use were the largest drug category (n=27, 12.74%), followed by drugs for the nervous system (n=24, 11.32%) and the cardiovascular system (n=23, 10.85%) (Multimedia Appendix 2).

Theoretically, 20,706 possible combinations could be formed from these 212 drugs; however, only 3463 drug combinations (16.7%) were documented in at least one of the data sources in this study. The summary of drug compatibility data extracted from the 3 reference sources is shown in Multimedia Appendix 3. Among the documented drug combinations, a total of 1653 (47.7%) had at least one record of incompatibility. Furthermore, 135 drug combinations showed conflicting compatibility results (ie, were listed as both compatible and incompatible) depending on the different data sources or specific trial conditions outlined in the ASHP’s Handbook. In total, 5416 distinct interaction records were identified. This number is higher than the number of drug combinations because some combinations, particularly in the ASHP’s Handbook, had multiple data items corresponding to different trial conditions. The initial interrater agreement for the database extraction between the two clinical pharmacists was excellent, with a Cohen Kappa of 0.91. All identified discrepancies were fully resolved through consensus-building before final integration.

### Network Visualization System for Multidrug Compatibility Query

**Figure 1.**
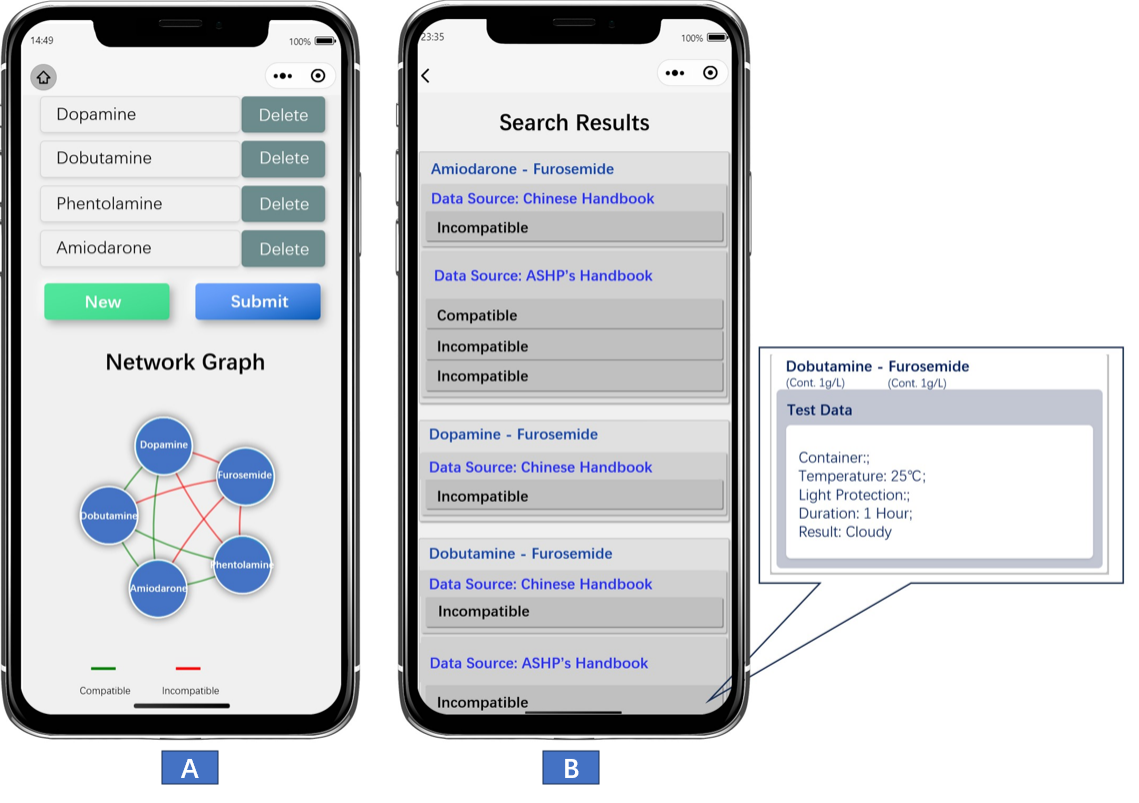
User interface of the network visualization query system. (A) The network graph interface, where nodes represent drugs and edges represent compatibility status (green for compatible, red for incompatible). (B) The detailed search results panel, providing text-based compatibility information from each data source and access to specific experimental conditions.

Our system was comprised of three main components: a drug combination input module, a network visualization interface, and a detailed search results panel. Users could input an unlimited number of drugs for analysis. The compatibility relationships were presented as a network graph generated by ECharts.js, where each drug was represented as a node. The edges connecting the nodes indicated the compatibility status, where a green edge indicated that the drug combination was documented as compatible across all data sources, a red edge indicated an incompatibility reported in at least one source, and the absence of an edge signifies a lack of available data. A drug combination was classified as “Compatible” only if supported by at least one source with no conflicting incompatibility data (Figure 1A). The search results panel provides a text-based list detailing the compatibility information for each drug combination from different data sources. By clicking the result button, users could access further details, such as the experimental conditions (Figure 1B).

### Participant Demographics

**Figure 2.**
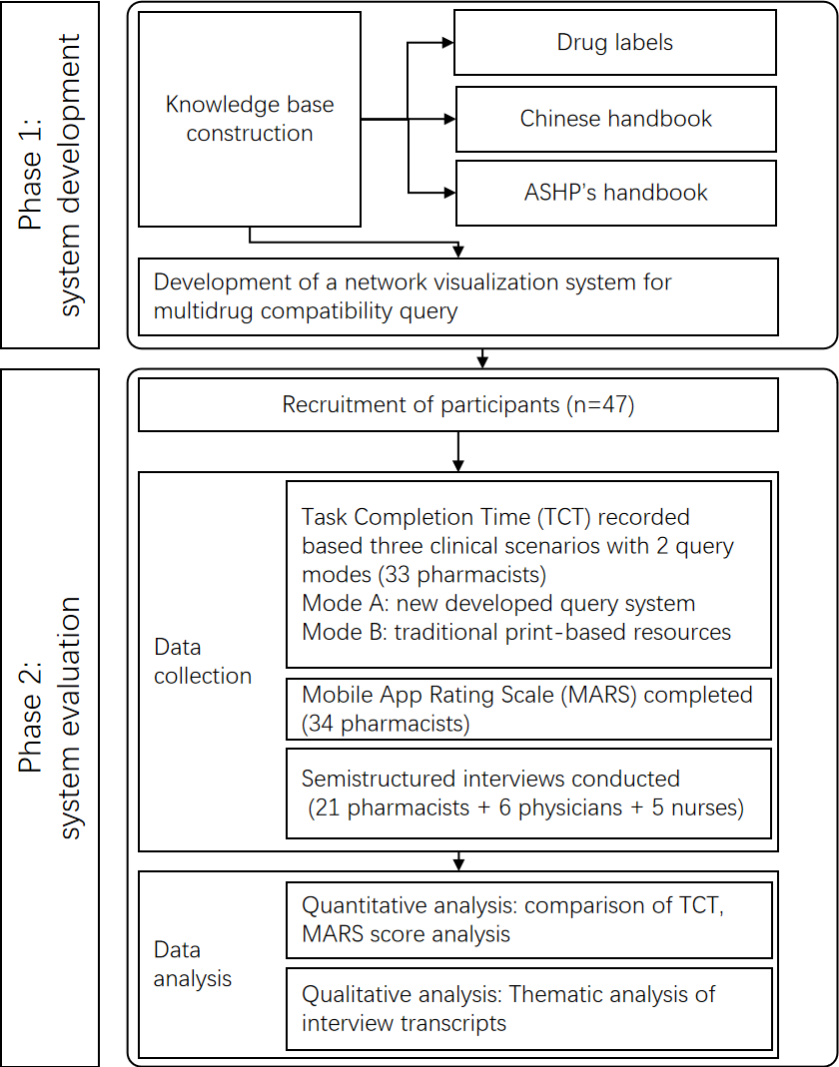
Flowchart of the preliminary crossover evaluation and posttask structured feedback design. The study was conducted in 2 phases. Phase 1 involved the development of the knowledge base and the query system. Phase 2 comprised the system evaluation, including data collection (task completion time, Mobile App Rating Scale assessment, and semistructured interviews) and data analysis.

A total of 47 health professionals from 12 medical institutes participated in the study (Figure 2). Among the 36 invited pharmacists for the quantitative phase, 34 successfully enrolled and completed the MARS assessment, while 2 dropped out entirely due to unexpected clinical emergencies during their scheduled testing slots. Out of the 34 enrolled pharmacists, 33 completed the crossover task completion time analysis (1 participant was unable to complete the time test due to urgent personal affairs but completed the MARS). For the subsequent interviews, 22 pharmacists participated voluntarily, and were joined by 5 physicians and 5 nurses who were additionally invited. Participants primarily worked in Tertiary A-level hospitals (39/47, 83%), with a mean age of 35.8 (SD 7.1) years and an average of 12.3 (SD 7.4) years of work experience. The majority held a bachelor’s degree (24/47, 51.1%). Detailed demographics for each study were presented in Table 1.

**Table 1.** Demographic characteristics of study participants.

Characteristics	All participants (n=47)	Task completion subgroup (n=33)	MARS^a^ subgroup (n=34)	Interview subgroup (n=32)
Number of unique institutions	12	9	10	9
Age (years), mean (SD)	35.8 (7.1)	34.5 (7.3)	34.8 (7.4)	35.9 (5.8)
Work experience (years), mean (SD)	12.3 (7.4)	10.7 (6.9)	11.2 (7.4)	12.3 (7.2)
Sex, n (%)				
Female	29 (61.7)	20 (60.6)	22 (64.7)	20 (62.5)
Male	18 (38.3)	13 (39.4)	12 (35.3)	12 (37.5)
Education level, n (%)				
Bachelor’s degree	24 (51.1)	15 (45.5)	17 (50)	17 (53.1)
Graduate degree	14 (29.8)	9 (27.3)	8 (23.5)	12 (37.5)
Associate degree	7 (14.9)	7 (21.2)	7 (20.6)	3 (9.4)
Vocational school	2 (4.3)	2 (6.1)	2 (5.9)	—^b^
Professional role, n (%)				
PIVAS^c^ pharmacist	15 (31.9)	14 (42.4)	15 (44.1)	6 (18.8)
General pharmacist	10 (21.3)	10 (30.3)	10 (29.4)	7 (21.9)
Clinical pharmacist	7 (14.9)	7 (21.2)	7 (20.6)	6 (18.8)
Physician	6 (12.8)	—	—	6 (18.8)
Nurse	5 (10.6)	—	—	5 (15.6)
Inpatient pharmacist	2 (4.3)	1 (3)	2 (5.9)	1 (3.1)
TCM^d^ clinical pharmacist	1 (2.1)	—	—	1 (3.1)
Prescription-reviewing pharmacist	1 (2.1)	1 (3)	—	—
Hospital level, n (%)				
Tertiary A-level	39 (83)	25 (75.8)	26 (76.5)	26 (81.2)
Nursing home	4 (8.5)	4 (12.1)	4 (11.8)	4 (12.5)
County-level	2 (4.3)	2 (6.1)	2 (5.9)	2 (6.2)
Community hospital	2 (4.3)	2 (6.1)	2 (5.9)	—
Department, n (%)				
Pharmacy	37 (78.7)	33 (100)	34 (100)	22 (68.8)
Neonatology	3 (6.4)	—	—	3 (9.4)
PICU^e^	2 (4.3)	—	—	2 (6.2)
Pediatric surgery	2 (4.3)	—	—	2 (6.2)
ICU^f^	1 (2.1)	—	—	1 (3.1)
NICU^g^	1 (2.1)	—	—	1 (3.1)
Surgery	1 (2.1)	—	—	1 (3.1)

a MARS: Mobile Application Rating Scale.

b Not available.

c PIVAS: pharmacy intravenous admixture service.

d TCM: traditional Chinese medicine.

e PICU: pediatric intensive care unit.

f ICU: intensive care unit.

g NICU: neonatal intensive care unit.

### Task Completion Time Analysis

**Figure 3.**
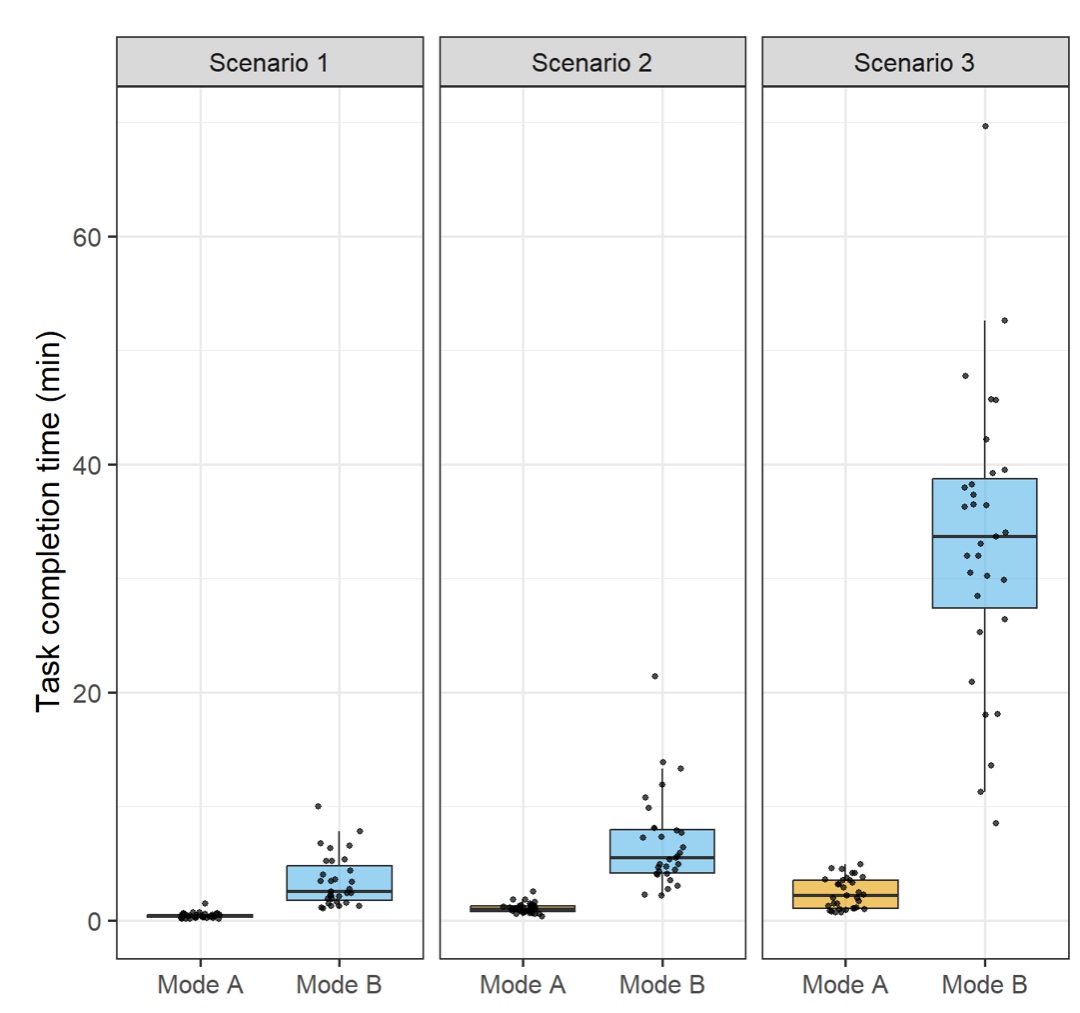
Boxplot with individual data points showing task completion time (in minutes) for the query system (Mode A) and traditional print-based resources (Mode B) across the three clinical scenarios. The box represents the IQR, the horizontal line indicates the median, and each dot represents an individual participant’s completion time.

The query system (Mode A) demonstrated a statistically significant and substantial reduction in task completion time compared to traditional resources (Mode B) across all scenarios (Figure 3). The median time savings were 2.85 (IQR 1.98‐4.15) minutes for Scenario 1 (V=555, *r*=0.85; *P*<.001), 5.45 (IQR 3.95‐7.20) minutes for Scenario 2 (V=545, *r*=0.82; *P*<.001), and 31.2 (IQR 27.5‐35.1) minutes for Scenario 3 (V=551, *r*=0.84; *P*<.001). Wilcoxon rank-sum tests confirmed the absence of significant order effects for all scenarios (*Ps_1_*=.43, *Ps_2_*=.67, *Ps_3_*=.42), validating the crossover design (Sequence 1 [Mode A first]: N_A_=12; Sequence 2 [Mode B first]: N_B_=21), though this unequal sequence allocation (12 vs 21) (Multimedia Appendix 4) warrants a cautious interpretation due to potentially limited statistical power in detecting carryover effects.

### MARS Score

The system received a high mean overall quality score of 3.88 (SD 0.35) on the MARS. Functionality scored the highest (mean 4.21, SD 0.51), while engagement scored the lowest (mean 3.21, SD 0.62). As shown in Table 2, the overall objective quality demonstrated excellent internal consistency (*α*=0.87; 95% CI 0.81‐0.92). The functionality (*α*=0.78) and aesthetics (*α*=0.77) subscales met the conventional 0.70 reliability threshold [30]. In contrast, the engagement (*α*=0.61), information (*α*=0.67), and subjective quality (*α*=0.48) subscales yielded values below this 0.70 threshold.

**Table 2.** Descriptive statistics and Cronbach α (n=34).

Subscale	Mean (SD)	Range	Cronbach α (95% CI)
Engagement	3.21 (0.62)	2‐4.60	0.61 (0.45‐0.79)
Functionality	4.21 (0.51)	2.75‐5	0.78 (0.69‐0.87)
Aesthetics	3.75 (0.56)	3‐5	0.77 (0.60‐0.88)
Information	3.86 (0.43)	2.80‐4.60	0.67 (0.53‐0.82)
Overall quality	3.76 (0.44)	2.84‐4.67	0.86 (0.81‐0.92)
Subjective quality	3.79 (0.52)	2.25‐4.75	0.48 (0.25‐0.71)

### Correlation and Generalizability Analysis

Spearman correlation analysis was conducted to examine the relationships between user background, usability ratings, and efficiency gains. No significant correlation was found between the MARS overall quality score and the objective time savings in any scenario (eg, Scenario 3: rs =0.11, *P*=.58), nor between MARS scores and user demographics such as age (rs =0.021, *P*=.92), or work experience (rs =0.11, *P*=.58). Regarding efficiency, while a significant positive correlation was observed between age and time savings in the simplest task (Scenario 1: rs =0.45, *P*=.01), efficiency gains in the most complex task (Scenario 3) were independent of age, experience, or education (all *P*>.05).

### Posttask Structured User Feedback

#### Overview of Descriptive Feedback

Descriptive analysis of the posttask structured feedback revealed five major areas of user experience with the multidrug compatibility query system. These areas were: (1) baseline experience and first impressions; (2) user experience with network visualization; (3) perceived efficiency and cognitive load; (4) trust, information quality, and adoption willingness; and (5) future application and implementation considerations. A summary of the themes, subthemes, and illustrative codes is presented in Table 3.

**Table 3.** Categorized user feedback, subcategories, and descriptive examples related to the user experience of the multidrug compatibility query system.

Feedback area and subcategory	User feedback examples
Baseline experience and first impressions	
Preexisting workflow challenges	Time-consuming Incomplete information Conflicting sources
Initial system impressions	Efficient and convenient Clear and intuitive Skepticism about data accuracy
User experience with network visualization	
Cognitive and comprehension support	Intuitive red and green lines Visualizing multiple relationships at once Aligning with natural thought process
Design feedback	“No connection” is ambiguous Suggesting a third color for conflicting data Needing clearer visual hierarchy
Perceived efficiency and cognitive load	
High cognitive load in traditional workflow	Memorizing drug pairs Repetitive manual searching Integrating scattered information
Reduction in mental effort	Eliminates need for memorization One-click comprehensive results Streamlines complex scenarios
Trust, information quality, and adoption	
Confidence and verification need	Cautious adoption (80%‐90% confidence) Double-checking for high-risk cases Needing to verify unfamiliar results
Trust-building elements	Transparent data sources Links to original evidence Explanations for incompatibility (the “why”)
Future application and implementation	
Feature iteration suggestions	Add management advice or alternatives Expand drug database (oral, TCM)^a^ Improve search function (eg, by Chinese pinyin)
High-value application scenarios	ICU^b^ bedside use PIVAS^c^ Emergency department decisions
Foreseen implementation barriers	Lack of brand recognition Data update and maintenance concerns Cost and institutional adoption

a TCM: traditional Chinese medicine.

b ICU: intensive care unit.

c PIVAS: pharmacy intravenous admixture service.

#### Area 1: Baseline Experience and First Impressions

This section of feedback encapsulates participants’ preexisting challenges and their initial perceptions of the Mini Program. A strong consensus existed across all professions that the traditional workflow for checking multidrug compatibility is inefficient, fragmented, and often inconclusive, posing a significant challenge in daily clinical practice.

The drug label doesn’t explicitly state its compatibility with other drugs. This means we have to consult additional resources, and we can’t always provide timely answers.

[Pharmacist, Participant 7]

The biggest challenge is the time it takes. You have to search through a lot of materials, and sometimes by the time you find the answer, the clinical window to use the drug has already passed.

[Physician, Participant 4]

The challenge for us is when there’s an urgent situation. We spend a lot of time searching, and if we can’t find the information, we get very anxious.

[Nurse, Participant 16]

Upon using the Mini Program, the first impression was overwhelmingly positive across the board, with all user groups praising its speed, clarity, and convenience.

Wow, it’s efficient, convenient, and fast. That was my most profound first impression.

[Pharmacist, Participant 20]

The first impression is that it is very concise and the interface is designed clearly. Even as a first-time user, I could get started easily without much familiarization.

[Physician, Participant 4]

It’s very convenient, which saves a lot of time, especially when you are busy or in the middle of a critical rescue.

[Nurse, Participant 9]

Despite this positive reception, an undercurrent of professional skepticism regarding data reliability was a dominant initial reaction, a concern articulated by all professions.

The downside is that for someone new to the app, there’s a sense of distrust; you don’t know if its data is professional.

[Pharmacist, Participant 3]

The advantage is definitely its convenience... The disadvantage is that we still have to reserve some doubt about its authenticity. I’m not sure if it’s really true, just like how AI can fabricate sources.

[Physician, Participant 5]

#### Area 2: User Experience With Network Visualization

This section of feedback focuses on participants’ interaction with the core network graph feature. The visualization was widely lauded for its intuitiveness.

It’s very intuitive. The red line directly shows you that this combination is incompatible.

[Pharmacist, Participant 15]

The use of red and green lines is very intuitive. The red light, green light model is quite intuitive.

[Nurse, Participant 16]

However, the interpretation of an absence of a connecting line was a significant point of confusion and divergence. This ambiguity was a common concern, leading to different risk assessments across roles.

I think a prompt should be added, because without a staff member nearby, it might not be clear.

[Pharmacist, Participant 1]

The confusion is that some drugs are not connected. I’m not sure what that means. Maybe we just won’t combine them and will pay a bit more attention to avoid putting them together.

[Physician, Participant 24]

For drugs like vancomycin that have no connecting lines to many others, I would probably choose to infuse it separately to be safe.

[Nurse, Participant 9]

This difference in interpretation led to a strong consensus on the need for improvement: an explicit label or a different line style to clearly indicate “insufficient data.”

#### Area 3: Perceived Efficiency and Cognitive Load

This section of feedback delves into how the query system changed the workflow and mental effort. When using traditional methods, participants identified information searching and integration as the most mentally demanding tasks.

Searching... When you are dealing with six drugs, my mind just goes blank trying to remember which one I’ve checked.

[Pharmacist, Participant 3]

I think it’s information integration. Because when there are too many drugs, it’s easy to get confused.

[Physician, Participant 4]

It’s both searching and integrating. You search and search, and you forget what you found earlier.

[Nurse, Participant 13]

The Mini Program was reported to significantly reduce this cognitive load, a game-changer for all professions.

It saved me the process of flipping through so many books. The efficiency has improved a lot.

[Pharmacist, Participant 4]

The cognitive burden is significantly reduced. In the past, you had to remember the last drug you checked. Now, you just input everything at once, and the system shows you the integrated result.

[Physician, Participant 6]

#### Area 4: Trust, Information Quality, and Adoption Willingness

This section of feedback addresses the crucial factors influencing user confidence. A pattern of cautious trust emerged, where most users would perform a secondary check under specific circumstances.

I think I would adopt it 80%‐90% of the time. But if I’m very certain that it should be compatible, I might check other resources.

[Pharmacist, Participant 1]

Because it’s a new program and I don’t know who developed it or where the data comes from, I won’t trust it without hesitation for now.

[Physician, Participant 5]

If the system shows a conflict for two drugs, I might think back to my clinical experience. If I’ve never encountered it, I will trust the result.

[Nurse, Participant 9]

To enhance trust, a universal demand was for transparent, accessible, and authoritative data sources.

The best way to build trust is to let us know the reason for incompatibility. If it provides a description, like precipitation or turbidity, it would be much more persuasive when we communicate with clinicians.

[Pharmacist, Participant 12]

The suggestion is... for example, when it shows compatibility or incompatibility, provide more data. I saw that it had sources, but we couldn’t click to read the in-depth information.

[Physician, Participant 5]

When asked to choose between information detail and query speed, the overwhelming consensus was that accuracy and detail are paramount.

I think both are very important... you need to be fast, but the accuracy of the query must also be guaranteed. After all, you are responsible for the patient.

[Pharmacist, Participant 1]

Definitely accuracy. The accuracy of information is the most important.

[Physician, Participant 6]

#### Area 5: Future Application and Implementation Considerations

This final section of feedback gathers participants’ suggestions. The most frequently requested feature was the provision of management advice, moving the tool toward a true decision support system.

After it gives an ’incompatible’ conclusion, could it provide some alternative solutions or management advice?

[Pharmacist, Participant 3]

It would be good to provide some clinical guidance, for example, how far apart I should administer them.

[Physician, Participant 24]

Participants identified high-stakes clinical environments as the most valuable scenarios.

I think this tool is most valuable in settings like the ICU, anesthesiology, and the IV compounding center, where multiple drugs are used simultaneously.

[Nurse, Participant 19]

The most valuable scenario is during prescribing in the ICU. It would be best if it could be embedded into our EHR system.

[Physician, Participant 21]

Finally, lack of official endorsement and potential cost were seen as the biggest barriers to widespread implementation.

I think the main barrier is building its reputation. If it doesn’t have a good reputation, nobody will know about this product.

[Pharmacist, Participant 1]

The obstacle? Maybe getting approval from the leadership. And whether it costs money.

[Nurse, Participant 25]

## Discussion

### Principal Findings

In this study, we found that a network visualization query system showed promising improvements over traditional, print-based methods for multidrug compatibility checking within this pilot evaluation. By combining objective, crossover usability testing with structured posttask feedback, we observed a substantial and statistically significant reduction in task completion time alongside high ratings for overall quality and functionality on the MARS scale. Crucially, the inclusion of a diverse sample of pharmacists, physicians, and nurses captured a comprehensive view of the system’s potential within a real-world clinical setting. This multiprofessional perspective was highly valuable for identifying divergent workflow needs. While clinicians universally praised the system’s efficiency and intuitive interface, their willingness to adopt was heavily contingent on the transparency and authoritativeness of the underlying data sources. Participants expressed a strong need for features that build trust, such as direct links to evidence and clear explanations for incompatibility, underscoring that successful clinical decision support implementation may benefit from tailored outputs that support the distinct needs of each professional group within the collaborative care process.

### Outcome Interpretation

In China, the drug label was the first-line reference for compatibility information due to its legal authority, a practice that differs from other countries where ASHP’s Handbook is considered the gold standard [31-33]. However, our study found that across all scenarios, the drug label contained the least amount of information on drug compatibility, a finding consistent with the common issue of data scarcity for drug compatibility in clinical practice, as evidenced by a study in a Chinese obstetrics and gynecology hospital where drug compatibility was a primary issue in telephone drug consultations [34]. Another fact is that drug incompatibility had been one category of off-label uses [35], exposing clinicians to significant legal risks without statutory protection, which further complicated the situation. Authoritative resources such as ASHP’s Handbook could be an alternative solution, but the English-language barrier prevents its widespread adoption in many clinical settings in China. Consequently, to address this dilemma created by a high demand for reliable compatibility data, insufficient legally sanctioned primary reference, and inaccessibility of a Chinese version of ASHP’s Handbook, our system, which integrates multiple data sources, is therefore a potential strategy to address this gap.

Our knowledge base construction revealed that 135 drug combinations showed conflicting compatibility results across different sources. This highlights another critical challenge in clinical practice: data inconsistency. Our system addresses this by transparently presenting data from all sources and flagging incompatibilities if reported in at least one source. This ‘safety-first’ approach empowers clinicians to make more informed decisions by being aware of the existing evidence conflicts, rather than being misled by a single, potentially incomplete data source.

The comparative results of task completion time analysis demonstrated that our query system was substantially more efficient than traditional methods, which were often time-consuming and prone to omission. Across all scenarios, our system enabled pharmacists to rapidly obtain drug compatibility information through easy operations, regardless of their experience or knowledge. Interestingly, while efficiency gains in the highly complex scenario (Scenario 3) were independent of demographic variables, a significant positive correlation between age and time savings was observed in the simpler Scenario 1. This stands in sharp contrast to traditional methods for query efficiency. When relying on drug labels, pharmacists not only face the challenge of reviewing large volumes of text, which can easily lead to overlooking important compatibility data, but they also encounter informational inconsistencies. This suggests that older pharmacists, who may be physically slower at manually cross-referencing thick paper-based books, experienced a more pronounced relative benefit from the digital tool. This aligns with prior research by Manrique-Rodríguez et al [19], which observed that the search order in one drug combination affected the results. In the Lexicomp database, a search for “verapamil” yields compatibility data with “vancomycin,” whereas a reverse search for “vancomycin” provides no such information. Relying on a single-directional query can lead pharmacists to mistakenly believe that compatibility data is unavailable, thereby compromising the accuracy and safety of their clinical decisions. To overcome these challenges, our system performs a bidirectional cross-validation search, extracting data from both drugs’ respective sources. Subsequently, the algorithm summarizes and deduplicates the compatibility information, offering the user comprehensive compatibility data. This process resolves the issue of data omission caused by search order or reliance on a single source and, therefore, ensures the completeness and reliability of the results. Additionally, this substantial improvement in efficiency is not merely a matter of time savings; it directly translates to a reduction in extraneous cognitive load. By automating the burdensome tasks of searching, memorizing, and integrating disparate information, the system alleviates the cognitive burden on clinicians. This allows them to focus on higher-order clinical reasoning and patient-specific factors, which, as cognitive load theory suggests, is crucial for reducing the risk of error [36] and enhancing patient safety [37].

On the MARS scale, our system achieved a high score in the functionality dimension. This result differed from a system review of web-based pharmacy apps [27] in which the aesthetics dimension achieved the highest score (mean 3.62, SD 0.50), followed by functionality. One of the reasons might be that, compared to our system, most commercial apps were developed by large companies where staff were professionally trained in user interface development. Additionally, our system was an early-stage prototype focused on functionality. In contrast, commercial apps were mature products that had been optimized through user feedback. Furthermore, because the engagement, information, and subjective quality subscales did not meet the strict 0.70 reliability benchmark, their corresponding scores must be interpreted with caution, potentially due to the small number of items [38]. The lower internal consistency observed for the MARS subjective quality dimension appears to stem from participants’ divergent views on the system’s value proposition, particularly regarding willingness to pay. Qualitative feedback revealed a clear dichotomy. One group of pharmacists viewed the tool as an essential institutional responsibility, arguing that such a critical safety utility should be provided by the hospital without charge to the individual. This perspective, while acknowledging high functionality, led to lower subjective scores related to personal cost. Conversely, another group perceived the significant time savings and reduced cognitive load as a direct personal and professional benefit that justified a purchase. They framed the app as an investment in their own workflow efficiency and well-being. This highlights that for clinical decision support tools, subjective value is complex, influenced not only by usability but also by perceptions of professional roles and institutional obligations. Future adoption strategies should likely prioritize institutional subscription models over individual payment to align with the expectations of most clinicians.

The inclusion of physicians and nurses in our qualitative analysis revealed critical interprofessional differences in how the system’s utility is perceived, underscoring the need for role-specific adaptations. Pharmacists, as primary users, valued the tool for its comprehensive and authoritative information retrieval capabilities. In contrast, physicians and nurses viewed it more as an action-oriented decision aid, expressing a greater need for immediate clinical guidance, such as management recommendations for incompatibilities or suggestions for alternative drugs. Furthermore, while pharmacists found the mobile format convenient, physicians advocated for integration with desktop EHR systems where prescribing occurs, and nurses highlighted the practical challenges of using smartphones at the busy bedside. These divergent perspectives on utility, workflow integration, and the interpretation of missing data emphasize that successful implementation requires a multiplatform strategy with tailored outputs that support the distinct needs of each professional group within the collaborative care process.

### Other Self-Developed Drug Compatibility Tools

Several studies have reported self-developed drug compatibility tools. Wang et al [39] designed a system named PharmDE for evaluating drug-excipient compatibility. This system constructed a database containing 532 incompatibility data items from 228 articles and established 60 drug-excipient interaction rules, providing functions such as database searching, data matching by similarity, and risk evaluation for both single drugs and formulations. In addition to such complex systems, simpler tools like tables or charts have been implemented in many hospitals. A study in a Japanese ICU [40] developed a drug compatibility chart for 27 drugs, which achieved a high compliance rate and was shown to effectively prevent drug incompatibility incidents. Similarly, other studies [19,41] have developed compatibility charts for 47 and 78 commonly used IV drugs, respectively, for application in neonatal and pediatric ICUs. All those tools have been proven efficient in query speed; however, the results of the query remained in text format and were hard to display the relationship network among all the involved drugs. Additionally, traditional tables and charts are static and limited to pairwise comparisons. In a scenario involving more than two drugs, it still requires more time to query all the potential drug combinations.

The application of visualization techniques to clarify complex pharmacological data is a well-established method to solve this problem. Multiple studies have used such tools in managing multiple drug relationships and proven their efficacy. Zitnik et al [42] used network visualization to present how drugs are connected via shared targets and interaction patterns, enabling a clearer understanding of polypharmacy side effect mechanisms and supporting the interpretation of Decagon’s predictive modeling outcomes. Network visualization in Brattig’s study [43] mapped the complete set of known drug–drug interactions, with nodes sized by probability of interaction and edges colored by gender-specific relative risk. The approach highlighted key drugs and high-risk combinations, revealing interaction patterns and demographic disparities, and providing actionable insights for targeted interventions to reduce adverse drug reaction risks. In the DDInter database developed by Xiong et al [44], network visualization was a core feature for enhancing user experience and clinical decision-making, allowing clinicians to achieve the interaction profile at a glance and helping in quickly identifying high-risk combinations, and ultimately supporting safer prescribing.

As far as we know, there were few studies using network visualization to present complex compatibility relationships among multiple drugs. In this study, we used the visualization tool “ECharts.js,” which had been widely applied across diverse medical fields including neuroscience [45], oncology [46], precision surgery [47], rare diseases [48], and traditional Chinese medicine [49], to present complex biomedical data and relationships through interactive visualizations. By using network visualization, we transformed the query results into a dynamic network visualization. This can help users instantly identify all the compatibility information of involved drugs with diverse linkages connecting nodes.

### Limitations

Several limitations of this study must be acknowledged. First, the evaluation was conducted with a small, geographically localized sample in Xiamen, predominantly involving Tertiary A-level hospitals, which limits generalizability to grassroots medical institutions. Second, the knowledge base was constructed using Chinese-specific reference sources, which may limit the system’s immediate generalizability to other national health care settings with different formularies. Additionally, the lack of connecting lines (representing missing data) was sometimes misunderstood by users as “compatible,” posing a safety risk in high-risk settings. Future iterations will address this by introducing distinct visual cues, such as gray dashed lines or alerts, to clearly denote “No Data Available.” Furthermore, the current system lacks a severity classification for compatibility contraindications, which remains a key objective for future development. Finally, a fundamental constraint of this preliminary usability study is that no formal accuracy validation (eg, sensitivity, specificity, and false-negative testing of the compatibility database) was conducted against an independent clinical gold standard or laboratory assays. Because incorrect decision support outputs can lead to severe patient harm, this prototype is strictly intended for preliminary usability testing, and rigorous clinical accuracy validation is an absolute and mandatory precondition before any bedside clinical deployment is considered. Prospective clinical trials are warranted to validate the system’s direct impact on patient safety outcomes.

### Conclusions

This study demonstrates that a query system based on network visualization is a promising and valuable tool for clinical practice. It demonstrates potential to support the efficiency and usability of multidrug compatibility queries. By offering an easily understandable view of complex data, the system has the potential to support medication safety by mitigating cognitive load, which is a known risk factor for clinical errors. Future prospective trials are required to establish its direct impact on reducing clinical medication errors and its economic value.

## Supplementary material

10.2196/86583 Multimedia Appendix 1 Semistructured interview topic guide.

10.2196/86583 Multimedia Appendix 2 Distribution of injectable drugs included in the knowledge base according to the Anatomical Therapeutic Chemical (ATC) classification system.

10.2196/86583 Multimedia Appendix 3 Summary of drug compatibility data extracted from the three reference sources.

10.2196/86583 Multimedia Appendix 4 Task completion times of the query system stratified by sequence.

10.2196/86583 Checklist 1 CONSORT checklist.

## Abbreviations

ATC: anatomical therapeutic chemical
CONSORT: Consolidated Standards of Reporting Trials
CRBSI: catheter-related bloodstream infection
CVC: central venous catheter
EHR: electronic health record
ICU: intensive care unit
INN: international nonproprietary name
IV: intravenous
MARS: Mobile App Rating Scale
NICU: neonatal intensive care unit
PICC: peripherally inserted central catheter
PICU: pediatric intensive care unit
PIVAS: Pharmacy Intravenous Admixture Service
TCM: traditional Chinese medicine
WXML: Wei Xin markup language
WXSS: Wei Xin style sheets
