# Supplementary material for: A Network Visualization Query System for Multidrug Compatibility Based on a WeChat Mini Program: Preliminary Usability and Efficiency Evaluation
Source: JMIR Form Res. 2026 Jul 21;10:e86583. doi: 10.2196/86583 (PMC13388532; doi:10.2196/86583)
Supplement: Multimedia Appendix 1 [file formative-v10-e86583-s001.docx]

**Qualitative interview topic guide**

**1. Current challenges and first impressions**

**Challenges**:

In your daily work, what are the biggest challenges you face when handling multi-drug compatibility queries?

**Overall impression:**

After completing the tasks, what is your first impression of this Mini Program system?

What is the biggest difference compared to using traditional print handbooks?

**2. Network visualization experience**

**Comprehension and cognition:**

How did the network graph help you understand the compatibility relationships between multiple drugs?

Does the way it presents information align with your natural thought process?

Did it help you identify any relationships you might have overlooked during a manual search?

**Feedback on design elements:**

How intuitive are the "red/green lines" for indicating "incompatible/compatible"? Were there any points of confusion?

How do you interpret the drugs with no connecting lines? How important is this information to you?

Do you have any suggestions for improving the color scheme or graphic design?

**3. Efficiency and cognitive load**

**Process comparison:**

Thinking back to the complex scenario (6 drugs) with the print handbooks, which part of the process was the most mentally demanding? How did this process change when using the Mini Program?

**Functionality feedback:**

Could you specify which features or actions made you feel it was particularly efficient or easy to use?

In what ways did the system most significantly reduce your cognitive load?

Are there any operational steps or workflows that you feel could be further simplified?

**4. Trust and information quality**

**Adoption Willingness:**

When the system shows an "incompatible" result, how confident are you in directly accepting that conclusion?

Under what circumstances would you trust it without hesitation? When would you feel the need to secondarily verify the information?

**Trust-building elements:**

What additional information would enhance your trust in the system's conclusions?

Between the level of detail in the information and the speed of the query, which do you consider more important?

**5. Future application and suggestions**

**Feature iteration:**

If you were to design the next version, what is the most important feature you would add or modify?

**Scenario expansion:**

In which clinical scenarios do you see this tool being most valuable?

If this tool were to be used by nurses or physicians, what adjustments to its features or interface might be necessary?

Implementation Barriers: What barriers or resistance do you foresee in promoting this tool in a real-world clinical setting?
