# Supplementary material for: A Network Visualization Query System for Multidrug Compatibility Based on a WeChat Mini Program: Preliminary Usability and Efficiency Evaluation
Source: JMIR Form Res. 2026 Jul 21;10:e86583. doi: 10.2196/86583 (PMC13388532; doi:10.2196/86583)
Supplement: Multimedia Appendix 2 [file formative-v10-e86583-s002.docx]

**Distribution of injectable drugs included in the knowledge base according to the Anatomical Therapeutic Chemical (ATC) classification system.**

| **Level 1 of ATC code** | **Name** | **N** |
| --- | --- | --- |
| A | Alimentary tract and metabolism | 20 |
| B | Blood and blood forming organs | 17 |
| C | Cardiovascular system | 23 |
| D | Dermatological drugs | 1 |
| G | Genitourinary system and reproductive hormones | 6 |
| H | Systemic hormonal preparations, excluding reproductive hormones and insulins | 9 |
| J | Anti-infectives for systemic use | 27 |
| L | Antineoplastic and immunomodulating agents | 18 |
| M | Musculoskeletal system | 5 |
| N | Nervous system | 24 |
| R | Respiratory system | 6 |
| V | Various ATC structures | 8 |
|  | No ATC code | 40 |
