# Supplementary material for: A Network Visualization Query System for Multidrug Compatibility Based on a WeChat Mini Program: Preliminary Usability and Efficiency Evaluation
Source: JMIR Form Res. 2026 Jul 21;10:e86583. doi: 10.2196/86583 (PMC13388532; doi:10.2196/86583)
Supplement: Multimedia Appendix 3 [file formative-v10-e86583-s003.docx]

**Summary of drug compatibility data extracted from the three reference sources.**

| **Source** | **Drug Combinations** | **Interaction Records** | **Compatibility** | **Incompatibility** | **Unknown** |
| --- | --- | --- | --- | --- | --- |
| Drug Labels | 317 | 372 | 276 | 96 | 0 |
| Chinese Handbook | 3206 | 3206 | 1633 | 1573 | 0 |
| ASHP's Handbook | 480 | 1838 | 1408 | 410 | 20 |
