# Supplementary material for: A Network Visualization Query System for Multidrug Compatibility Based on a WeChat Mini Program: Preliminary Usability and Efficiency Evaluation
Source: JMIR Form Res. 2026 Jul 21;10:e86583. doi: 10.2196/86583 (PMC13388532; doi:10.2196/86583)
Supplement: Multimedia Appendix 4 [file formative-v10-e86583-s004.docx]

**Task Completion Times (Minutes) of the Query System (Mode A) Stratified by Sequence (n=33).**

| **Scenario** | **Sequence**  **(**Note: 1 = Mode A first; 2 = Mode B first.**)** | **SSample Size (n)** | **Task Completion Time (Minutes), Median (IQR)** |
| --- | --- | --- | --- |
| 1 | 1 | 12 | 0.30 (0.19 – 0.46) |
|  | 2 | 21 | 0.33 (0.22 – 0.52) |
| 2 | 1 | 12 | 1.00 (0.82 – 1.15) |
|  | 2 | 21 | 0.94 (0.60 – 1.15) |
| 3 | 1 | 12 | 2.00 (1.00 – 3.32) |
|  | 2 | 21 | 2.35 (1.03 – 3.50) |
