# Supplementary material for: A Network Visualization Query System for Multidrug Compatibility Based on a WeChat Mini Program: Preliminary Usability and Efficiency Evaluation
Source: JMIR Form Res. 2026 Jul 21;10:e86583. doi: 10.2196/86583 (PMC13388532; doi:10.2196/86583)
Supplement: Checklist 1 [file formative-v10-e86583-s005.docx]

**CONSORT Checklist**

| **Section/Topic** | **Item No.** | **CONSORT Checklist item** | **Reported on page No. and line No.** |
| --- | --- | --- | --- |
| **TITLE AND ABSTRACT** |  |  |  |
| Title | 1a | Identification as a crossover randomized trial in the title, or structured abstract. | Page 2, Line 11 (Abstract: Methods) |
| Abstract | 1b | Structured summary of trial design, methods, results, and conclusions. | Page 2, Lines 1-32 |
| **INTRODUCTION** |  |  |  |
| Background | 2a | Scientific background and explanation of rationale. | Page 3, Lines 1-38 |
| Objectives | 2b | Specific objectives or hypotheses. | Page 4, Lines 5-11 |
| **METHODS** |  |  |  |
| Trial design | 3a | Description of trial design (including crossover details, sequence allocation, washout period). | Page 4, Lines 13-18; Page 8, Lines 8-17 |
|  | 3b | Important changes to methods after trial commencement, with reasons. | None / Not applicable |
| Participants | 4a | Eligibility criteria for participants. | Page 7, Lines 4-10 |
|  | 4b | Settings and locations where the data were collected. | Page 7, Lines 4-6 |
| Interventions | 5 | The interventions for each period, including details of washout period (30-minute break) and query modes. | Page 8, Lines 4-17 |
| Outcomes | 6a | Completely defined pre-specified primary and secondary outcome measures. | Page 7, Line 19 - Page 8, Line 33 |
|  | 6b | Any changes to trial outcomes after the trial began, with reasons. | None / Not applicable |
| Sample size | 7a | How sample size was determined. | Page 7, Lines 6-10 (Purposive sampling) |
|  | 7b | When applicable, explanation of any interim analyses and stopping guidelines. | Not applicable |
| Randomization: |  |  |  |
| *Sequence generation* | 8a | Method used to generate the random allocation sequence. | Page 8, Line 15 ("via coin toss") |
|  | 8b | Type of randomization; details of any restriction (blocking, stratification). | Page 8, Lines 15-17 (Simple randomization) |
| *Concealment* | 9 | Mechanism used to implement the random allocation sequence. | Page 8, Lines 15-17 |
| *Implementation* | 10 | Who generated the random allocation sequence, who enrolled, and who assigned participants. | Page 24, Lines 22-27 (Authors' contributions) |
| Blinding | 11a | If done, who was blinded after assignment to interventions and how. | Not applicable (Unblinded due to study design) |
|  | 11b | If relevant, description of the similarity of interventions. | Not applicable |
| Statistical methods | 12a | Statistical methods used to compare outcomes between periods (Wilcoxon signed-rank test). | Page 8, Line 35 - Page 9, Line 12 |
|  | 12b | Methods for additional analyses, such as subgroup analyses and testing for carryover/order effects. | Page 9, Lines 1-7 (Wilcoxon rank-sum for carryover) |
| **RESULTS** |  |  |  |
| Participant flow | 13a | For each sequence group: numbers of participants randomly assigned, receiving intended treatment, and analyzed. | Page 11, Lines 9-14 |
|  | 13b | For each period: losses and exclusions, with reasons. | Page 11, Lines 11-14 |
| Recruitment | 14a | Dates defining the periods of recruitment and follow-up. | Page 7, Line 2 ("May 2025 to September 2025") |
|  | 14b | Why the trial ended or was stopped. | Completed planned schedule |
| Baseline data | 15 | A table showing baseline demographic and clinical characteristics for each sequence group. | Page 11-12, Table 1 |
| Numbers analyzed | 16 | For each group, number of participants included in each analysis and whether analyzed by original assigned groups. | Page 11, Lines 12-14 |
| Outcomes and estimation | 17a | For each primary and secondary outcome, results for each group/period, and estimated effect size. | Page 13, Lines 1-10; Page 14, Table 2 |
|  | 17b | For binary outcomes, results of absolute and relative effect sizes. | Not applicable |
| Ancillary analyses | 18 | Results of any other analyses performed, including subgroup analyses (correlation with demographics). | Page 14, Lines 12-20 |
| Harms | 19 | All important harms or unintended effects in each group. | None reported / Not applicable |
| **DISCUSSION** |  |  |  |
| Limitations | 20 | Trial limitations, addressing sources of potential bias, imprecision, and carryover concerns. | Page 23, Lines 5-23 |
| Generalizability | 21 | Generalizability (external validity, applicability) of the trial findings. | Page 23, Lines 5-11 |
| Interpretation | 22 | Interpretation consistent with results, balancing benefits and harms, and considering other relevant evidence. | Pages 19-22 (Discussion) |
| **OTHER INFORMATION** |  |  |  |
| Registration | 23 | Registration number and name of trial registry. | Not registered (Institutional ethics approved: Page 9, Lines 25-30) |
| Protocol | 24 | Where the full trial protocol can be accessed, if available. | Available from corresponding author upon request |
| Funding | 25 | Sources of funding and other support, role of funders. | Page 25, Lines 2-4 (Funding declaration) |
